# Estimating Community Drug Abuse by Wastewater Analysis

**DOI:** 10.1289/ehp.11022

**Published:** 2008-05-01

**Authors:** Ettore Zuccato, Chiara Chiabrando, Sara Castiglioni, Renzo Bagnati, Roberto Fanelli

**Affiliations:** Department of Environmental Health Sciences, Istituto di Ricerche Farmacologiche Mario Negri, Milano, Italy

**Keywords:** amphetamines, cannabis, cocaine, drug residues, illicit drugs, mass spectrometry, opiates, sewage epidemiology, urinary metabolites

## Abstract

**Background:**

The social and medical problems of drug abuse are a matter of increasing global concern. To tackle drug abuse in changing scenarios, international drug agencies need fresh methods to monitor trends and patterns of illicit drug consumption.

**Objective:**

We tested a sewage epidemiology approach, using levels of excreted drug residues in wastewater, to monitor collective use of the major drugs of abuse in near real time.

**Methods:**

Selected drug target residues derived from use of cocaine, opiates, cannabis, and amphetamines were measured by mass spectrometry in wastewater collected at major sewage treatment plants in Milan (Italy), Lugano (Switzerland), and London (United Kingdom). The amounts of drug residues conveyed to the treatment plants, reflecting the amounts collectively excreted with urine, were used to estimate consumption of the active parent drugs.

**Results:**

Reproducible and characteristic profiles of illicit drug use were obtained in the three cities, thus for the first time quickly revealing changes in local consumption (e.g., cocaine consumption rose significantly on weekends in Milan). Profiles of local drug consumption based on waste-water measurements are in line with national annual prevalence estimates.

**Conclusions:**

Patterns and trends of drug abuse in local communities can be promptly monitored by this tool, a convenient new complement to more complex, lengthy survey methods. In principle, searching the sewage for excreted compounds relevant to public health issues appears to have the potential to become a convenient source of real-time epidemiologic information.

Official figures for the prevalence and occurrence of drug abuse in different countries are currently obtained from population surveys integrated with crime statistics, medical records, and drug production and seizure rates [European Monitoring Centre for Drugs and Drug Addiction (EMCDDA) 2006; United Nations Office of Drugs and Crime (UNODC) 2007a]. These statistical tools give a useful general picture of drug abuse, but estimates of consumption rates and drug use prevalence may be inaccurate, as most of the information is obtained from the consumers themselves. Moreover, because data collection and analysis are time consuming, current methods cannot offer frequently updated results to quickly detect changing patterns, and it is not easy to compare results between local communities (EMCDDA 1997). As recently recommended by the Commission of Narcotic Drugs of the United Nations (UNODC 2007b), novel approaches are needed not only to provide more realistic and comparable estimates of illicit drug consumption in different communities, but also to detect changes in drug abuse patterns rapidly.

We tested a sewage epidemiology strategy to monitor patterns and trends of community-wide illicit drug use. The idea of using drug monitoring at sewage treatment plants (STPs) to assess collective drug consumption was presented as a speculative proposal by Daughton in 2001, and the methodologic approach was devised and implemented by our group using cocaine as a model drug in 2005 (Zuccato et al. 2005). Sophisticated analytical methods for measuring several drugs of abuse have since been set up by us and others, as reviewed by Castiglioni et al. (2008).

Urban wastewater entering an STP is an accessible, economical source of real-time, pooled epidemiologic information (Bohannon 2007). Human metabolic waste products that are rapidly collected and pooled by the sewage systems can provide valuable evidence of the amount and type of any common product consumed by a population (Dove 2006). Earlier work with therapeutic drugs has shown close correspondence between the known amounts consumed by the population and amounts estimated from concentrations of metabolic drug residues measured in wastewater (Heberer and Feldmann 2005; Lindberg et al. 2005). If an excretion product is stable in wastewater and efficiently conveyed to the STP, it is reasonable to assume that the amount collectively excreted in a given period should be reflected by the amount reaching the STP in the corresponding interval (Daughton 2001). Therefore, we sought to provide objective, quantitative, near-real-time profiles of illicit drug consumption by monitoring selected drug residues entering the municipal sewage system. To test this approach, we chose a panel from among the most-used illicit drugs worldwide (Table 1), selecting their main excretion products as analytical targets for wastewater monitoring (Castiglioni et al. 2006).

## Materials and Methods

### Drugs of abuse

Community-wide consumption of common drugs of abuse, that is, cocaine, heroin, cannabis, and amphetamine-type drugs [amphetamine, methamphetamine, ecstasy (3,4 methylenedioxymethamphetamine)] was estimated by analysis of selected drug excretion residues in wastewater.

### Selection of drug target residues

The drug residues targeted for wastewater measurement and back-calculation of drug consumption are referred to as drug target residues (DTR). An ideal DTR is a major and exclusive excretion product (metabolite or unchanged parent drug) of the drug under study that is stable in wastewater. The DTRs used for this study (Table 1) were chosen by determining the metabolic fate of each active drug in light of current knowledge and then experimentally determining the stability of candidate residues in wastewater (Castiglioni et al. 2006). We thus selected as DTRs the main urinary metabolites for cocaine, heroin, and cannabis, and the unchanged parent drug for the amphetamines (Baselt 2004; Huestis et al. 1996; Maurer et al. 2006). Glucuronic acid–conjugated metabolites, the most abundant excretion products of heroin and cannabis, had to be discounted as DTRs because of their instability in wastewater. Glucuronates are rapidly hydrolyzed back to the corresponding free compound by fecal bacterial enzymes (D’Ascenzo et al. 2003; Ternes et al. 1999). Our stability studies (Castiglioni et al. 2006) confirmed that glucuronic acid–conjugated metabolites added to wastewater rapidly disappear, releasing the free compound. Therefore, the selected DTRs for heroin and tetrahydrocannabinol (THC) were free morphine and 11-nor-9-carboxy-Δ9-tetrahydrocannabinol (THC-COOH).

### Wastewater sampling

Composite samples of untreated urban wastewater were collected from the inlet of the STPs by sampling waste-water every 20 min for 24 hr in a time-proportional mode, then pooling the subsamples with an automatic, computer-controlled device. To test the reproducibility over time of field data from a given STP, samples were taken on consecutive days for 1 week on three different occasions in Milan, Italy (Nosedo STP, sampled for 7 days in November 2005 and in February and March 2006; population served, 1.25 million), and for 1 week in Lugano, Switzerland (STP sampled for 7 days in March 2006; population served, 0.12 million). Measurements were also taken in London, United Kingdom, at two major plants (Mogden and Beckton STPs, sampled for 2 days in October 2005; populations served, 1.8 and 3.7 million).

### Analysis of DTRs in wastewater

Illicit drug residues (Table 1) were measured in wastewater samples with a fully validated, highly selective multiresidue assay described in detail by Castiglioni et al. (2006). Briefly, water samples were spiked with internal standards, acidified, and solid-phase extracted on mixed reversed-phase/cation-exchange cartridges (Oasis-MCX; Waters Corp., Milford, MA, USA), preconditioned with methanol, water, and 0.01N HCl, then eluted with methanol and 2% ammonia in methanol. The pooled eluates were analyzed by liquid chromatography–tandem mass spectrometry using an API 3000 triple quadrupole mass spectrometer, equipped with a turbo ion spray source (Applied Biosystems–Sciex, Thornhill, Ontario, Canada) interfaced to LC Series 200 pumps and an autosampler (Perkin-Elmer, Norwalk, CT, USA). Drugs were analyzed using an XTerra MS C18, 100 mm × 2.1 mm, 3.5-μm column (Waters Corp.) at a flow rate of 200 μL/min. Quantitative analyses were done in the selected reaction monitoring (SRM) mode, measuring the fragmentation products of the protonated or deprotonated pseudomolecular ions of each compound and deuterated analog. The recoveries, repeatability, instrumental limits of detection (LODs), and limits of quantification (LOQs) for the entire method were calculated in wastewater samples as described (Castiglioni et al. 2006).

### From measured DTR concentrations to collective DTR excretion rates

Using the approach described for cocaine (Zuccato et al. 2005), the concentration (nanograms per liter) of a given DTR in wastewater was multiplied by the influent wastewater flow rate (liters per day) to calculate the amount of each DTR daily reaching a given STP (grams per day). The data were then normalized for the local population size (number of people served by the STP). Assuming no major loss of wastewater along the sewage system and given the proven stability of the chosen DTR in wastewater (Castiglioni et al. 2006), these figures (milligrams per day per 1,000 people) reasonably reflect the collective excretion rates for the various DTRs.

### From DTR excretion rates to collective drug consumption rates

The collective excretion rate of a given DTR was used to extrapolate the amount of the active parent drug consumed by the population under study. This was done by correcting the amount of each excreted DTR by a factor (Table 1), taking into account the known fraction of the consumed parent drug normally excreted as DTR in urine, and the parent drug-to-DTR molar mass ratio (Table 1). For example, about 45% of intranasal cocaine (molecular weight 303) is excreted in urine as benzoylecgonine (BE; molecular weight 289), so a measured BE excretion rate of 100 mg/day/1,000 people corresponds to 100/0.45 × 303/289 = 233 mg of cocaine consumed per day per 1,000 people.

These calculations are valid when a DTR is a main specific excretion product of a single parent drug and therefore a reliable direct indicator of consumption. This applies to all the DTRs used here except morphine, a residue not excreted solely after intake of heroin, but after morphine and codeine as well. The fraction of wastewater morphine originating from consumed codeine was considered negligible, as morphine is a minor metabolite of codeine (Baselt 2004). When back-calculating heroin consumption based on wastewater morphine, corrections were therefore applied only to compensate for the obviously substantial contribution from therapeutic morphine (Ther-M). Briefly, we first considered the known average consumption of Ther-M in Italy, Switzerland, and the United Kingdom: 11, 82, and 123 mg morphine/day/1,000 people, respectively (Zuccaro et al. 2006). The daily amounts of wastewater morphine expected to originate from Ther-M, back-calculated from these figures, were then subtracted from the total daily amounts of wastewater morphine. The remaining wastewater morphine was assumed to originate mostly from heroin.

### From drug consumption rates to the number of doses consumed

To compare our estimates of collective drug consumption with official figures that mainly refer to drug use prevalence, we had to translate total consumed amounts of parent drugs into the corresponding number of average consumption units (i.e., doses), as defined by official statistics. The actual amount of pure drug in a consumption unit is not easily determined because drugs of abuse can be taken by various routes in amounts that vary widely among different consumer groups and in different phases of an individual’s history of use (Cohen and Sas 1994). In addition, the purity of street products fluctuates unpredictably with time and in different locations, leading to possible miscalculation of the actual average amounts of active drug taken as a dose. Despite these limitations, however, for each drug we established a best-approximation average dose on the basis of the literature and official statistics. The average content of pure active drug in a typical dose taken by the most common route (UNODC 2004) was assumed here to be approximately 100 mg for intranasal cocaine, 30 mg for oral amphetamine and methamphetamine, 100 mg for oral ecstasy, 30 mg for intravenous heroin, and 125 mg for smoked THC (based on high-potency cannabis: 14% THC in hashish/marijuana). The number of doses consumed daily in the three cities was then calculated by dividing drug consumption rates (milligrams per day per 1,000 people) by these amounts of active principle in an average dose.

## Results

### DTR excretion data as objective indices of drug consumption

**Figure 1:**
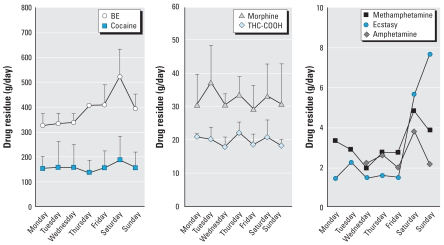
Average daily amounts (mean ± SD, g/day, *n* = 3) of illicit drug residues conveyed by wastewater to Milan’s STP (1.25 million people served). Levels of amphetamines were near or below the LOD based on available data (2-week period). To allow a rough comparison with the profiles of the other, more abundant drugs, undetectable levels were considered 50% of the limit of quantification (LOQ; typically around 1 ng/L in wastewater).

The total daily amounts of DTRs reaching an STP directly reflect the collective excretion of these residues by an undetermined number of drug consumers in the population served by that plant. Figure 1 shows the average daily amounts of DTRs reaching Milan’s STP, serving 1.25 million people. On average, about 0.5 kg BE (a major cocaine metabolite), 200 g cocaine, 40 g morphine (in part derived from heroin), 25 g THC-COOH (main residue from cannabis), and a few grams of amphetamines reached the plant every day. When DTR excretion rates are normalized for the number of people served by the plant (milligrams per day per 1,000 people), they can be compared between different communities (Tables 2 and 3).

### Reproducibility of DTR excretion data

From the repeated weekly surveys in Milan, the average daily collective DTR excretion rates appeared reproducible, for major, steadily detectable DTRs, between different days and weeks [relative standard deviation (RSD) < 16% over 7 days and < 19% over 3 weeks for BE, THC-COOH, and morphine] (Table 4). When replicate data grouped according to the day of the week were analyzed by one-way ANOVA, collective excretion of most DTRs appeared generally steady over time, with a significant peak of BE from cocaine on Saturdays (*p* < 0.01 vs. Monday, *p* < 0.02 vs. Tuesday or Wednesday, Dunnett’s test). There was a non-significant increase during the weekend for the amphetamines. Morphine and THC-COOH remained constant during the week (Figure 1), suggesting steady use of heroin and cannabis in Milan. Variation was also limited for most major DTRs in Lugano and London as well (Table 2).

### Estimated illicit drug consumption

**Figure 2:**
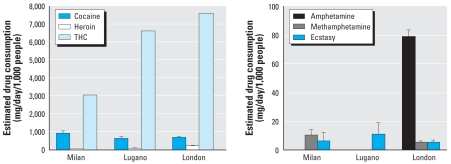
Estimated consumption rates (mean ± SD, mg/day/1,000 people) of illicit drugs in Milan, Lugano, and London, back-calculated from DTR excretion rates after correction for the factors shown in Table 1. Estimates for amphetamine-type drugs are shown only where DTR levels were measurable (in > 85% samples). Estimates of heroin consumption were back-calculated after subtracting the fraction of wastewater morphine presumably excreted as a product of therapeutic morphine, as expected from the known morphine consumption in the three countries.

Further processing of DTR excretion data allowed us to back-calculate consumption rates (milligrams per day per 1,000 people) for the illicit drugs, as described in “Materials and Methods” and Table 1. Results for the three cities (Figure 2) show similarity in the consumption profiles of cannabis, cocaine, and heroin (THC >> cocaine > heroin). Our data suggest that people in Milan tend to consume slightly more cocaine and less cannabis than people in Lugano and London (Tables 2 and 3).

For the amphetamine-type drugs, the picture was more complex and harder to interpret because their residues in wastewater were generally low and often undetectable, in line with recent findings in Spain (Huerta-Fontela et al. 2007). Nevertheless, it was evident that amphetamine consumption was much higher in London than in Milan, despite similar methamphetamine use in these cities. The use of ecstasy, the only amphetamine-type drug detected in Lugano, did not differ substantially in the three cities.

### Estimated heroin consumption

**Figure 3:**
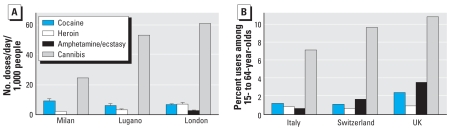
Comparison of local profiles of illicit drug use (mean ± SD, doses/day/1,000 people) obtained from drug residues in wastewater and national profiles of drug use (defined as the percentage of users among persons 15–64 years of age) based on annual prevalence data in the countries under study. (*A*) Data derived from estimated drug consumption rates (Figure 2) divided by the amount of the active drug in a typical dose. Values for amphetamine/ecstasy that are barely visible are 0.42 ± 0.18, 0.11 ± 0.08, and 2.9 ± 0.2 doses/day/1,000 people in Milan, Lugano, and London, respectively. (*B*) Data from the UNODC (2006).

With all the caveats regarding the use of morphine as a DRT for heroin, we first assessed whether the contribution of heroin use to wastewater morphine was indeed substantial. We therefore additionally monitored a minor (possibly fluctuating) but exclusive metabolite of heroin, 6-acetylmorphine. The presence of 6-acetylmorphine in the wastewater samples from Milan and Lugano, at levels that averaged about 5% of measured morphine (data not shown), proved that heroin steadily contributes to wastewater morphine. The estimates of heroin consumption shown here (Figures 2 and 3) for Milan, Lugano, and London were then back-calculated from total wastewater morphine, after subtracting the fraction presumably originating from local therapeutic use of morphine (Table 5). Wastewater morphine from heroin consumption accounted for about 70, 30, and 40% in Milan, Lugano, and London, respectively. Accurate, updated information about local morphine use should therefore be considered in future studies to refine the assessment of actual heroin consumption.

### Comparison of wastewater-derived data with official statistics

We next verified whether our estimates were in line with official epidemiologic data describing the drug abuse phenomenon. We compared local profiles of illicit drug use (defined as number of doses per day per 1,000 people), obtained from measurement of drug residues in wastewater, with national profiles of drug use (defined as the percentages of users among persons 15–64 years of age) obtained from annual prevalence data in the countries under study. Despite the limitations arising from the necessary assumptions in defining average consumption units (i.e., doses), our approach gave local drug use profiles (Figure 3A) in line with patterns of drug use based on national annual prevalence data (Figure 3B) (UNODC 2006), except for amphetamine-type drugs.

## Discussion

Our evidence-based approach for monitoring collective illicit drug use gave reproducible and comparable profiles. Repeated weekly monitoring of DTRs of Milan STP on different occasions showed that the method can detect significant fluctuations in consumption that consistently occur during a week, such as the rise in cocaine use toward the weekend (Figure 1). As expected, drugs that tend to be consumed steadily, such as cannabis, have stable DTR excretion along the week, with only small variations on different occasions (Figure 1).

Comparison of DTR excretion data from Milano, Lugano, and London (Table 2) offered direct evidence that the three profiles of drug use have many similarities and a few local peculiarities in line with local drug use habits, for example, high amphetamine consumption in the United Kingdom (UNODC 2007a, 2007b). These findings suggest that wastewater measurements provide objective, direct evidence of collective DTR excretion that can be used to compare patterns of illicit drug use in different communities.

Our drug use profiles (number of daily doses per 1,000 people) agree with official annual prevalence figures in indicating that the drug most used by far is cannabis (Figure 3). The relative importance of cocaine and heroin use is similarly represented by the two methods. However, our use profiles, but not prevalence estimates, suggest that amphetamines (including ecstasy) are the drugs least used in all locations (Figure 3). A possible reason may lie in the intrinsic differences in the two approaches. Our sewage approach offers direct evidence of relative consumption rates, but no indication about the number of users. Prevalence figures, on the other hand, focus on the number of users, often without specific reference to use patterns (e.g., number of doses per month, occasional or continuous use) or the amounts consumed (e.g., size of personal doses). As the number of users is generally defined as the percentage of people (often within specific age groups) who admit having used a drug in a given interval (e.g., the last month), prevalence data may tend to overestimate the use of drugs that are used occasionally by many (e.g., the amphetamines) rather than used steadily by a few.

Despite this latter limitation, we tried to assess how our approach compared with official data in terms of number of doses used in these populations. For example, for cocaine, national prevalence figures (EMCDDA 2006) indicate that in Italy 1.2% of adults (15–64 years of age)—about 10,000 people in Milan—used the drug during the last year. If these people were all light users of cocaine (consuming, on average, 16 g/person/year) (Caulkins et al. 1999), they would collectively consume about 160 kg/year. Our figures, however, provide direct evidence that about 330 kg of cocaine are used in the city each year, suggesting that the actual amount consumed by the predicted users in Milan is higher than the light use standard. If most cocaine consumers in Milan were light users, more people would be involved than expected from national prevalence figures. This example suggests that our estimates compare reasonably with official figures, while offering sound evidence of overall drug consumption that could be used to refine and integrate official statistics, especially at the local level.

Our sewage approach to drug consumption monitoring has three main advantages. The first, and most important, is the use of objective, quantifiable measures (i.e., DTR concentration, wastewater flow rate, population size) providing realistic and reproducible pictures of the amount and type of illicit drugs consumed in different communities (Table 6). Another point is that these results can be obtained in near real time, because wastewater drug profiling by multiresidue mass spectrometric analysis can be completed in 1 or 2 days after sampling. The third benefit comes from the possibility of integrating wastewater data with other information on illicit drug use (e.g., metabolism/kinetics, average doses, purity of street products) to refine the estimates of drug consumption and improve comparability of drug use profiles. This data integration is only feasible by defining assumptions based on best current knowledge (Table 6).

Given that this is a newly implemented approach, we offer our critical view of the potential biases in Table 6 and list a number of actions that may be taken in future studies to improve the accuracy of the calculations and assumptions used here. A multifaceted critical assessment seems desirable with a view to improving the current approach on the basis of joint expertise from researchers, local authorities, and international drug agencies. Concerted actions could be aimed in particular at *a*) locally controlling parameters in the sewer system and population size fluctuations in the STP catchment area; *b*) integrating collective consumption data with updated statistics on local drug use patterns for each drug (e.g., intake routes, frequency of use, size of typical dose); and *c*) refining the assumptions related to metabolism/kinetics of the various drugs by further experimental work and mathematical modeling. Considering that our first unrefined approach to monitoring cocaine use by waste-water analysis (Zuccato et al. 2005) is already being applied in different countries, including the United States (Bohannon 2007; Bones et al. 2007), a consensus view appears essential for the comparability of future studies.

In conclusion, testing wastewater for illicit drug residues provides objective field data that can offer a reliable picture of collective drug residue excretion in a large community. Data can be further elaborated with quantitative assumptions to estimate the consumption of the active principle for the various drugs and the overall number of daily doses consumed. The sewage epidemiology approach to drug consumption monitoring could be used prospectively for *a*) using updated drug profile analyses to rapidly identify emerging hot spots of drug abuse; *b*) testing in real time the efficacy of different countermeasures such as prevention through education, enforcement, and global concerted actions against illicit drug consumption; *c*) cross-validation of population surveys versus wastewater monitoring programs; and *d*) assessing the actual amount of illegal money involved in drug trafficking.

If applied to other public health issues, this approach has the potential to extract useful epidemiologic data from qualitative and quantitative profiling of biological indicators entering the sewage system.

## Figures and Tables

**Table 1:** Analytical targets (DTR) selected for illicit drug monitoring in wastewater.

Drug	DTR	Relation of DTR to parent drug	Percentage of drug dose excreted as DTR^a^	Molar mass ratio (parent drug/DTR)	Correction factor
Cocaine	BE	Major metabolite	45	1.05	2.33
	Cocaine	Parent drug (minor excretion product)			
Heroin	Morphine	Major but nonexclusive metabolite	42	1.29	3.07
	6-Acetylmorphine	Minor but exclusive metabolite			
Amphetamines
Amphetamine	Amphetamine	Parent drug and major excretion product	30	1.0	3.3
Methamphetamine	Methamphetamine	Parent drug and major excretion product	43	1.0	2.3
Ecstasy	Ecstasy	Parent drug and major excretion product	65	1.0	1.5
Cannabis	THC-COOH	Major metabolite of THC (cannabis active principle)	0.6	0.91	152

Levels of DTRs were used for back-calculating drug consumption; the correction factor takes into account the percentage of parent drug excreted as the chosen DTR, and the parent drug-to-DTR molar mass ratio.

a Average for the most frequent route of intake.

**Table 2:** Amounts (mg/day/1,000 people) of major DTRs from illicit drug consumption conveyed daily in urban wastewater to STPs in Milan, Lugano, and London.

DTR	Milan	Lugano	London (Mogden; Becton)^a^
BE	390 ± 63	267 ± 52	296 ± 18 (302; 290)
Cocaine	157 ± 14	109 ± 23	140 ± 10 (141; 139)
Morphine	32 ± 3	102 ± 15	173 ± 29 (196; 150)
THC-COOH	20 ± 2	43 ± 10	50 ± 21 (56; 44)

Values reflect collective DTR excretion rates. Data are mean ± SD of daily samplings for 1 week and 3 nonconsecutive weeks at Milan STP (*n* = 21), and for 1 week at Lugano STP (*n* = 7). Two London STPs were sampled for 2 days, on Thursday and Friday (*n* = 4).

a Values in parentheses represent averages for Mogden and Beckton STPs, respectively.

**Table 3:** Amounts (mg/day/1,000 people) of DTRs from amphetamine-type drugs conveyed daily in urban wastewater to STPs in Milan, Lugano, and London.

DTR	Milan^a^	Lugano	London
Amphetamine	2.7 ± 2.8 (5/14)	ND (0/7)	24 ± 5 (4/4)
Methamphetamine	4.5 ± 1.6 (14/14)	ND (0/7)	2.4 ± 0.3 (4/4)
Ecstasy	4.2 ± 3.7 (12/14)	7.3 ± 5.1 (7/7)	3.4 ± 1.0 (4/4)

ND, not detectable. Values in parentheses represent the number of positive/total samples. Data are mean ± SD, with values for negative samples averaged as half the LOQ.

a Data for amphetamine-type drugs were available for 2 weeks from Milan.

**Table 4:** Variation in collective excretion rates (mean ± SD, g/day) of major DTRs between days (RSD for the average of seven daily means) and between weeks (RSD for the average of three weekly means) in Milan.

	Collective excretion of DTR (g/day)										
DTR	Monday (*n* = 3)	Tuesday (*n* = 3)	Wednesday (*n* = 3)	Thursday (*n* = 3)	Friday (*n* = 3)	Saturday (*n* = 3)	Sunday (*n* = 3)	Average of daily means (*n* = 7)	Average of weekly means (*n* = 3)	Between-days variation (RSD, %)	Between-weeks variation (RSD, %)
BE	326 ± 47	334 ± 41	338 ± 35	407 ± 7	409 ± 80	522 ± 111	394 ± 60	390 ± 63	390 ± 39	16	10
Cocaine	153 ± 49	158 ± 104	157 ± 93	136 ± 51	155 ± 70	188 ± 94	155 ± 65	157 ± 14	157 ± 74	9	47
Morphine	31 ± 9	37 ± 11	31 ± 3	34 ± 5	29 ± 7	33 ± 10	31 ± 12	32 ± 2	32 ± 6	8	19
THC-COOH	21 ± 1	20 ± 4	18 ± 3	22 ± 3	19 ± 3	21 ± 5	18 ± 2	20 ± 1	20 ± 2	7	11

**Table 5:** Back-calculation of heroin consumption (mg/day/1,000 people) in Milan, Lugano, and London after correcting for the contribution of therapeutic morphine to the overall amount of wastewater morphine.

	Milan	Lugano	London
Therapeutic morphine consumption^a^	11	82	123
Estimated excretion of Ther-M^b^	9	70	105
Total-M	32	102	173
Heroin-derived morphine	23	32	68
Back-calculated heroin consumption	70	100	210

Total-M, total morphine measured in wastewater. Heroin-derived morphine = Total-M – Ther-M.

a Based on yearly consumption of morphine in Italy, Switzerland, and United Kingdom of 4, 30, and 45 mg per capita per year (Zuccaro et al. 2006).

b Back-calculated from consumption rates, taking into account the DTR fractional excretion (85%).

**Table 6:** Characteristics, advantages, and potential limitations of the “sewage approach” for monitoring illicit drug consumption.

			Possible bias				
Measurements	Type of data	Reliability of data	Source of bias	Probability of occurrence	Estimated inaccuracy (this study)	Action to improve accuracy (future large-scale studies)	
Excretion of DTR (mg/day/1,000 people)	Concentration of DTR in wastewater (ng/L)	Potentially very reliable (if validated, highly specific analytical methods are used)	Possible adsorption of some DTR to particulate	Low	Probably negligible	Monitoring multiple DTR for each drug Specific studies on DTR partition between water and particulate	
	Wastewater flow into STP (L/day)	Normally well controlled (in modern STP)	Leakage from sewers of substantial wastewater	Low	Probably low	Wastewater flow strictly controlled at STP, sewer leakage checked by dilution tests	
	Population size (no. of people served by STP)	Likely reliable (variations reflected by water consumption changes)	Fluctuating number of people in the catchment area (inhabitants, commuters, tourists, etc.)	Low to medium (depending on type of community)	Probably low (as proven by low variation over time of collective excretion rates for some DTR)	Actual number of people at any time in the catchment area monitored/controlled by various indicators (e.g., other human by-products in wastewater, energy consumption)	
				Possible bias			
Estimates	Type of information	Related assumptions	Source of information	Source of bias	Probability of occurrence	Estimated inaccuracy (this study)	Action to improve accuracy (future large-scale studies)

Drug consumption rate (mg/day/1,000 people)	Total fraction (%) of a drug dose excreted as DTR (used to back-calculate amount of drug consumed from amount of excreted DTR)	Definition of correction factors (Table 1) based on best available current knowledge Wastewater used as a surrogate pooled urine sample from local population Wastewater (24-hr sample) reflects near steady-state excretion rate of drug residues when STP serves large population (> 10^5^ people^a^) No significant fluctuation in drug use simultaneously in a large proportion of users	a. Current literature on human drug metabolism/kinetics b–c. Previous studies on therapeutic drugs showing correspondence between known drug consumption and back-calculated consumption (from wastewater DTRs)	Limited number of subjects in most studies	Low to medium (depending on drug under study)	Probably low for absolute rate of consumption if main specific metabolite is chosen No effect on comparability of drug use profile between locations over time at given location	Further studies on metabolism/kinetics for drugs of abuse (larger number of subjects, different consumption routes and use patterns). Meta-analysis of all available metabolism/kinetics studies
Number of doses consumed (no. of doses/day/1,000 people)	Amount of active drug in a typical dose (used to back-calculate number of doses from amounts consumed)	Definition of best approximated typical dose from available data	National/international drug agencies, official reports on drug abuse, scientific literature	Local differences in drug market Differences in drug intake route (intranasal, smoke, ingestion, injection) Different habits (light vs. heavy; regular vs. occasional use)	Variable Variable Variable	Can affect overall estimates of number of doses but not consumption rates Can affect estimated size of typical dose and fraction of drug dose excreted as DTR Can affect estimated size of typical dose	Local differences in drug market controllable by analysis of drugs seized Mathematical modeling to account for different patterns of drug intake (from consumer interviews) Mathematical modeling to account for drug use habits (from consumer interviews)

a Tentative estimate to be investigated in ad hoc studies.
